# Efficient liquid phase confiscation of nile blue using a novel hybrid nanocomposite synthesized from guar gum-polyacrylamide and erbium oxide

**DOI:** 10.1038/s41598-022-18591-0

**Published:** 2022-08-29

**Authors:** Daud Hussain, Suhail Ayoub Khan, Tabrez Alam Khan, Salman S. Alharthi

**Affiliations:** 1 Department of Chemistry, Jamia Millia Islamia, Jamia Nagar, New Delhi, 110 025 India; 2 Department of Chemistry, College of Science, Taif University, P.O. Box 110999, Taif, 21944 Saudi Arabia

**Keywords:** Chemistry, Materials science

## Abstract

In recent times, biopolymer-metal oxide nanocomposites have gained prominent importance in the attenuation of environmental toxicants from aqueous phase. But lanthanide oxide-based biopolymer nanocomposites have scantly been evaluated for their adsorption potential. A novel guar gum-polyacrylamide/erbium oxide nanocomposite (GG-PAAm/Er_2_O_3_ NC) adsorbent was synthesized by copolymerization of guar gum (GG) and acrylamide (AAm) utilizing *N*-*N*′-methylenebisacrylamide as a crosslinker and Er_2_O_3_ as a reinforcing agent. The adsorptive efficacy of GG-PAAm/Er_2_O_3_ nanocomposite was evaluated using nile blue (NB) as a model pollutant dye from aquatic system. The prepared adsorbent was characterized by Fourier transform infrared (FTIR) spectroscopy, X-ray diffraction (XRD) analysis, Brunauer–Emmett–Teller (BET) analysis, thermogravimetric analysis, scanning electron microscopy-energy dispersive X-ray spectroscopy (SEM–EDX), and high-resolution transmission electron microscopy (HRTEM). The optimal process parameters, which include dosage (0.8 g/L), agitation time (40 min), initial solution pH (6), and initial NB concentration (80 mg/L) were determined by batch methodology. The equilibrium data for NB confiscation was better expressed by Langmuir isotherm model, with maximal adsorption effectiveness (*Q*_*m*_) of 225.88 mg NB/g demonstrating the actively monolayer adsorption onto homogeneous surface of GG-PAAm/Er_2_O_3_ NC. The kinetics of NB sorption process onto GG-PAAm/Er_2_O_3_ NC was reliable with pseudo-second order model. Thermodynamic parameters such as Δ*H*^°^ (15–17 kJ/mol) and Δ*S*^°^ (0.079–0.087 kJ/mol/K), and − Δ*G*^°^ (8.81–10.55 kJ/mol) for NB validated the endothermic, an increased randomness at the GG-PAAm/Er_2_O_3_–NB interface, and spontaneity and feasibility of the process, respectively. The spent nanocomposite was effectively regenerated with NaOH, and could be reused proficiently for five runs demonstrating the high reusability potential of the nanocomposite. The commendable removal efficiency and high reusability of GG-PAAm/Er_2_O_3_ NC recommended it to be a highly competent adsorbent for cationic dyes particularly NB diminution from aqueous waste.

## Introduction

The control of environmental deterioration of aquatic resources resulting from the amplified discharge of liquid effluents due to rapid industrial development and global population growth has emerged as one of the major challenging tasks over the past several decades. Many industries such as textile, leather, tannery, cosmetics, paints or plastics are prime contributors of colored pollutants. The vast usage of synthetic dyes, which are mostly poorly-degradable and environmentally persistent due their complex molecular structures, in the textile industries emancipates huge quantity of dyes-laden water post dyeing and finishing operations. The release of unspent dyes in the receiving bodies not only worsens the water quality but presents a detrimental outcome including impaired photosynthesis of aquatic ecosystems, and carcinogenic, mutagenic or teratogenic influence on aquatic biota and humans^1–3^. Most dyes, on ingestion, are responsible for several diseases such as dizziness, vomiting, tremors, nausea, cyanosis and jaundice. Direct contact may result into allergic problems, skin irritation, and eye burn that can permanently damage the cornea. If inhaled, they may lead to breathing difficulty, profuse sweating, abdominal pain and hyper motility^2^. In the last few decades, much attention has been focused on mitigating the colored contaminants with a view to protecting the environmental sustainability and reduce the grave health risks associated with the industrial dyes^4,5^. Nile blue (NB) is an azo dye widely applied for dyeing in the textile industries. Many health-related problems including skin irritation, dermatitis, allergic reactions in eyes and respiratory disease are associated with its presence in water^6^. It can cause sleepiness, digestive system stimulation, cold feelings, mouth and throat irritation, redness and dryness of the skin, and chromosomal aberrations. Therefore, it is essential to decolorize the effluents containing the hazardous nile blue effectively before discharging into the aquatic system^7^.

The most common approaches for confiscating and decolorizing dyes from aquatic system are chemical coagulation, ozonolysis, photocatalysis, microbial degradation, electrochemical methods, and adsorption^8–10^. But most physico-chemical techniques are rather expensive, operationally cumbersome and energy-intensive. However, adsorption technology has attained widespread recognition as the most encouraging, reliable, highly effective, operationally simpler, efficient and cheap practice for environmental pollutants abatement with admirable regenerative and recyclable potential of the adsorbents.

Multitude of adsorbents such as activated carbons^10^, zeolites^11^, graphene oxides^12^, and clays^13^ have arduously been used for dyes decontamination. However, these adsorbents lack the traits of an ideal adsorbent, which has led to the pursuit for advanced adsorbents possessing higher sorption efficiencies. Over the recent years, biopolymers as adsorbents have gained popularity due to their environment-friendly characteristics^14^. However, due to their poor mechanical strength and fair water solubility, their usage in water pollution remediation is rather restricted. The utilization of metal oxides as filler in polymer matrices have minimized the strength related problem and has broadened their scope in water treatment. Metal oxides-based gum polysaccharide nanocomposites incorporating the synergistic assets of both the components have aroused a great deal of interest in aquatic environmental contaminants sequestration^15–17^ owing to their cheap and facile obtainability, feeble-toxicity, environmentally benign nature, improved surface area to volume ratio, easy modifiability, better biocompatibility, excellent mechanical strength, and magnificent adsorption effectiveness^17–20^. Many nanocomposite adsorbents derived from gum arabic^21^, gum karaya^22^, gum ghatti^23^, gum xanthan^24^, gum tragacanth^25^ have recently been described in the literature for sequestration of organic dyes from aquatic environment.

The modification of biopolymers is usually undertaken to impart certain functional groups/moieties to improve upon the surface characteristics and adsorptive performance. The nanocomposites based on guar gum (GG), a low-priced, innocuous, and biodegradable biopolymer, have shown promising applications as effective adsorbents for a number of organic and inorganic aquatic contaminants in the recent times^26–30^. However, to our best understanding, the adsorption studies on biopolymer/lanthanide oxide (nano)composites for the attenuation of diverse contaminants have hitherto received scant consideration. Recent researches on the removal of salicylhydroxamic acid and acid blue 92 by Nd_2_O_3_^31,32^, rhodamine B by r-GO/rare earth metal oxide aerogels^33^, reactive black 5 using LaFeO_3_/chitosan nanocomposite^34^, arsenite by carrageenan-embedded LaFeO_3_ nanocomposite^35^, and metal ions by ceria and its composites^36^ have been undertaken. Additionally, grafted/cross-linked GG composites, Ag NPs containing GG/poly(AA) grafted polymer^37^, guar gum grafted acrylic acid/nanoclay^38^, mGG-PAAm^39^, GG/magnetite/chitosan^40^, pectin-crosslinked-GG/SPION^41^, GG-crosslinked-graphene oxide hydrogel^42^ and GG-metal oxide (nano)composites, ZnO NPs/GG^43^, Fe_3_O_4_-GG^44^, and TiO_2_/GG hydrogel^45^ have been arduously employed for the confiscation of many environmental pollutants. However, adsorption studies invoking guar gum cross-linked with PAAm (polyacrylamide) using N, N-methylenebisacrylamide as a cross-linker and erbium oxide as the filler has not been attempted so far to the best of our knowledge. Further, its utilization as an adsorbent for nile blue is also undocumented.

The present investigation aims at developing a novel and recyclable nanocomposite adsorbent for organic dyes remediation based on erbium oxide-reinforced guar gum-polyacrylamide biopolymer matrix and to evaluate its adsorption competence towards an organic cationic dye. The synthesis of guar gum-polyacrylamide/erbium oxide nanocomposite (GG-PAAm/Er_2_O_3_ NC), its characterization through infrared and nuclear magnetic resonance spectroscopy, X-ray diffraction, N_2_ adsorption/desorption, and thermogravimetric analyses, and scanning and transmission electron microscopy, and its liquid phase removal efficacy for nile blue (NB) is depicted in the current study. The process variables (agitation time, initial solution pH, dosage, and initial NB concentration) impacting the NB removal was explored to ascertain the optimal operating conditions, and various facets of the adsorptive uptake phenomena was explicated in terms of isotherm and kinetic parameters deduced by applying non-linear regression analyses of the adsorption data using the corresponding model equations. Thermodynamics parameters were also examined to estimate the energetic changes accompanying the adsorption process. Several adsorption–desorption cycles were performed in order to estimate the reuse potential of the adsorbent.

## Materials and methods

### Chemicals and instrumentation

Guar gum (Loba chemie, India), Acrylamide (Spectrochem, India; 99.5%), *N*,*N′*-methylenebisacrylamide (MBA; Loba chemie, India; 99%), nile blue (Himedia, India; 96%), ammonium persulfate (APS) (Merck, India; 98%) were used as acquired without further purification. All chemicals were of AR standard. Double distilled water was employed for the preparation of working solutions. The operating pH of test solutions was adjusted between 2 and 10 by applying dilute HCl and NaOH solution (0.1 M). The FTIR spectra, XRD pattern, SEM images and TEM micrographs were obtained using Perkin-Elmer spectrometer, model BX spectrum, USA, Philips Analytica PW 1830 apparatus, (Philips, The Netherlands), Carl Zeiss JOEL scanning electron microscope, (Sigma 5.05, Germany) and Transmission electron microscope, HRTEM 200 kV model, FEI (Tecnai), respectively. TGA analyses (35 ^o^C–600^ o^C) were performed using a Perkin-Elmer thermal analyser (TGA 4000, Massachusetts, USA, Pyris 6 TGA with PyrisTM software V. 11.0.0.0449) at a heating rate of 10 ^o^C min^–1^ under nitrogen atmosphere. The initial and residual NB concentration was determined using a UV-visible spectrophotometer (T-80 + UV/Vis spectrophotometer, PG Instrument Ltd, UK).

### Synthesis of guar gum-polyacrylamide/Er_2_O_3_ (GG-PAAm/Er_2_O_3_) nanocomposite

The GG-PAAm/Er_2_O_3_ nanocomposite was prepared by cross-linking guar gum and acrylamide by employing MBA as a cross-linker in the presence of Er_2_O_3_ (filler). Aqueous suspension (100 ml) of guar gum (1.0 g), prepared by vigorous stirring for 1 h, and acrylamide solution (0.3 g in 10 mL of deionized water) were homogenously mixed under constant stirring at 60 °C followed by the addition of APS (1.0 g in 5 mL water) as initiator and Er_2_O_3_ as filler. *N*,*N′*-Methylenebisacrylamide (0.2 g) was added to the mixture under continuous stirring for 30 min, and the reaction mixture was sonicated for 1 h. The obtained product (%yield = 72) was washed with water and ethanol to remove unreacted moieties, oven-dried at 90 °C for 48 h, ground to a fine powder, and stored in a stoppered glass vial for further studies.

### Equilibrium sorption studies

The impact of different operating factors such as dosage, agitation time, initial solution pH, initial NB concentration, and temperature on removal efficiency of GG-PAAm/Er_2_O_3_ nanocomposite adsorbent was evaluated by batch adsorption experiments, wherein NB solution (50 mL) of known concentration (20, 30, 40, 50 mg/L) was agitated in a water bath shaker with desired adsorbent mass (0.2–1.2 g/L) for different contact time (10–60 min) at constant temperature. After agitating for the desired time interval, the solution was centrifuged and the [NB] in the supernatant was determined spectrophotometrically at maximum absorbance wavelength of 635 nm. Adsorption isotherm studies at different temperatures (303, 308, and 313 K) was accomplished by shaking NB solution of various concentrations (30–80 mg/L) till equilibrium was achieved. However, for kinetics appraisal, similar experiments were conducted with optimal nanocomposite dosage (0.8 g/L) at 303 K and pH 6 using varying [NB] (30, 40 and 50 mg/L) for optimal agitation time (40 min).

## Results and discussion

### Characterization of GG-PAAm/Er_2_O_3_ nanocomposite

**Figure 1:**
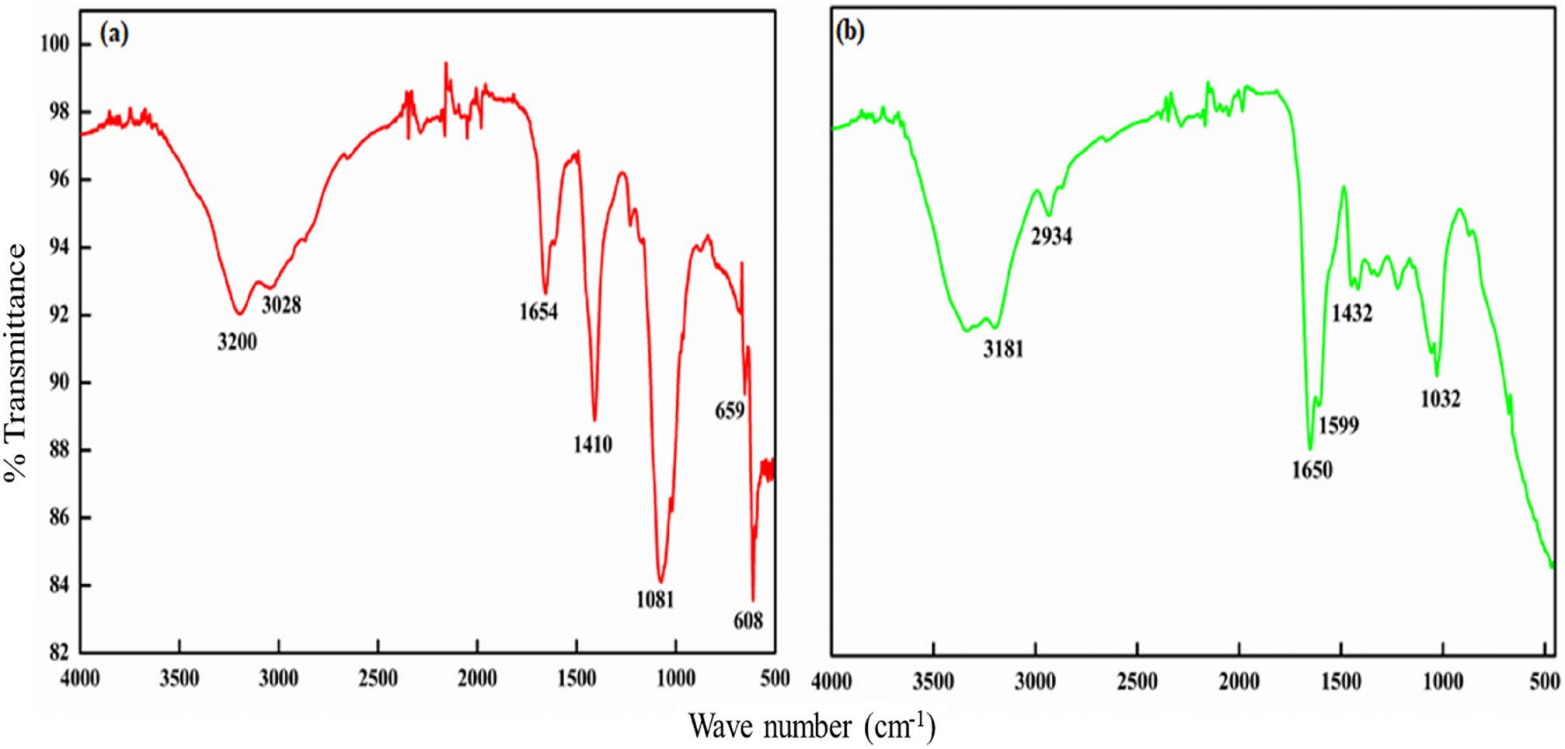
FTIR spectra of (**a**) GG-PAAm/Er_2_O_3_ nanocomposite, and (**b**) NB-loaded nanocomposite.

The FTIR spectra of GG-PAAm/Er_2_O_3_ nanocomposite, prior and post adsorption, is shown in Fig. 1. The diminished intensity of peaks around 3000–3500 cm^−1^ for GG-PAAm/Er_2_O_3_ NC signified the interaction of hydroxyl groups of guar gum with amide group of polyacrylamide^46^. The peaks detected in the spectra around 1200 cm^−1^ depicted the C–C–O, C–OH and C–O–C stretching modes of polysaccharides^14^. The peaks at 1654 cm^−1^ and 1604 cm^−1^ were due to C=O stretching vibrations in acrylamide^47^. The peak observed at 1081 cm^−1^ was attributed to bending vibration for CH_2_–O–CH_2_^48^, whereas the band at 1410 cm^−1^ due to C–N stretching vibrations^49^. The peak at 3028 cm^−1^ was ascribed to NH_2_ stretching vibrations of the polyacrylamide^50^. Additionally, the band at 659 and 608 cm^−1^ were attributed to Er–O–Er and Er–O linkage, respectively^51^, which clearly indicated the existence of metal–oxygen bond participating in the biopolymer nanocomposite. After NB adsorption (Fig. 1b), the FTIR spectrum showed slight shift in the peak position assigned to NH_2_ from 3028 to 3181 cm^−1^, and diminution of intensities together with slight shifting of absorption bands at 1654 and 1081 cm^−1^ to lower wavelengths indicating that the relevant functional groups were involved in the adsorption procedure either by van der Waals forces or hydrogen bonding^9^. Further, a slight shift in the O–H, C–O, and C–N bands; and a drop in the intensity of O–H stretching vibration at 3200 cm^−1^ after NB sorption, confirmed the interaction of dye molecules with existing functional groups^10^. Appearance of a new peak at 2934 cm^−1^ because of CH_2_ group of the aromatic ring structure of dye validated the sorption process onto the nanocomposite^52^.

**Figure 2:**
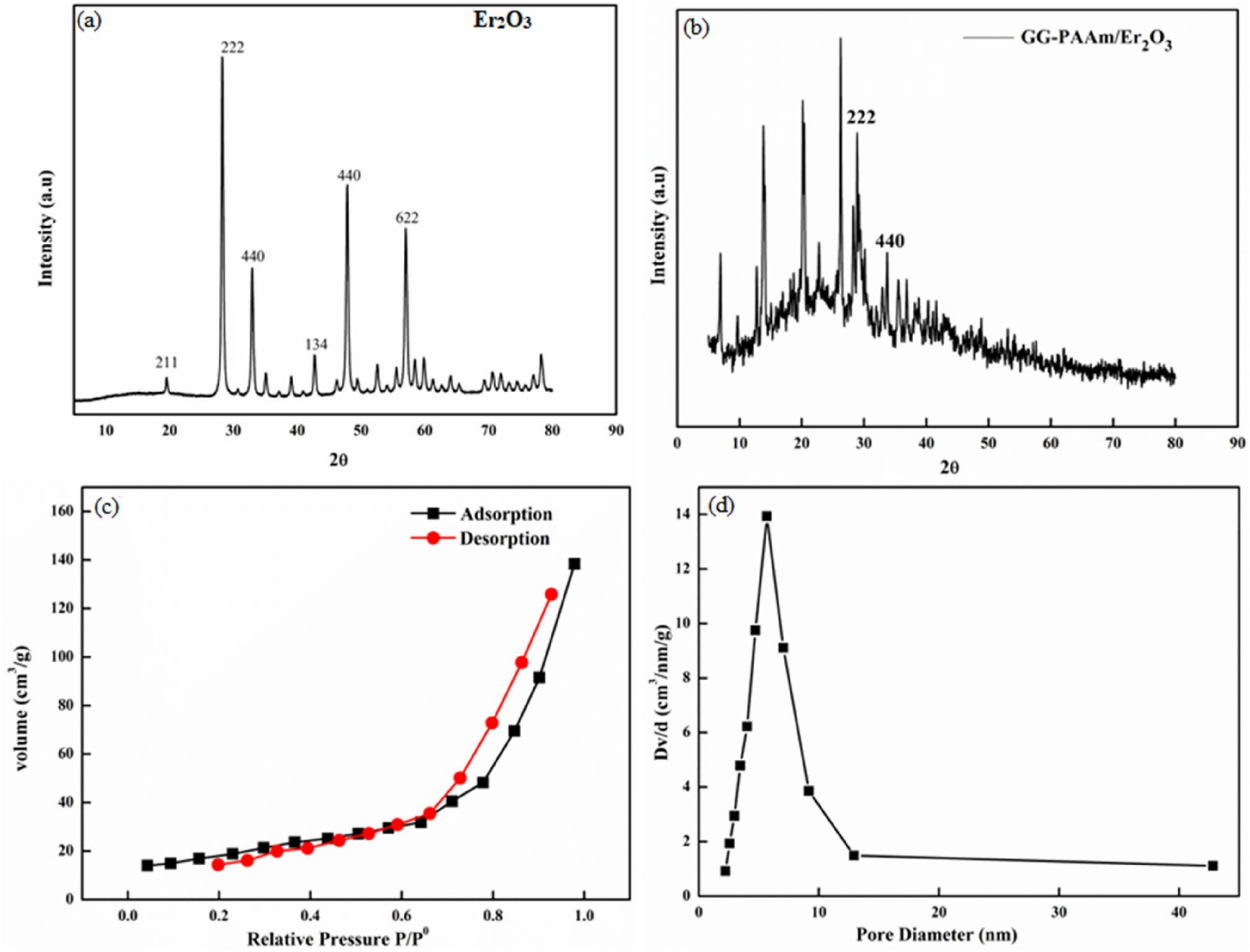
XRD spectra of (**a**) Er_2_O_3_, (**b**) GG-PAAm/Er_2_O_3_ nanocomposite, (**c**) N_2_ adsorption–desorption curve, and (**d**) pore size distribution.

The XRD diffraction pattern of Er_2_O_3_ is depicted in Fig. 2a that illustrated high crystallinity and sample purity. The main reflections of Er_2_O_3_ centred at 2ϴ = 19.56 (211), 28.60 (222), 32.82 (440), 42.72 (134), 48.54 (440), and 56.98 (622) matched completely with the JCPDS file no. 78–0390 having space group of Ia-3^53^. The existence of characteristic peaks for erbium oxide in the diffraction pattern along with some shifting of the diffraction peaks and the formation of a semi-crystalline network due to the amalgamation of Er_2_O_3_ into the amorphous guar gum phase signified the fabrication of GG-PAAm/Er_2_O_3_ NC (Fig. 2b). Average crystallite size of GG-PAAm/Er_2_O_3_ NC was 57 nm.

The N_2 _adsorption/desorption isotherm was used to determine the specific surface area and porosity of the nanocomposite. Higher surface area is related to greater accessibility of adsorptive sites, hence boosted adsorption aptitude of an adsorbent. The N_2 _adsorption/desorption isotherms of GG-PAAm/Er_2_O_3_ NC is shown in Fig. 2c,d. The specific surface area, pore volume and pore diameter of nanocomposite were 70 m^2^/g, 0.024 cm^3^/g and 5.796 nm, respectively. The pore diameter in the range of 2–50 nm confirmed the mesoporous nature of the prepared NC.

The thermal stability and rate of decomposition of the nanocomposite was gauged by TGA. This analysis could manifest the change in mass of the sample throughout the heating process. The TGA curves of GG and GG-PAAm/Er_2_O_3_ NC are shown in Fig. S1 revealing that the decomposition occurred in two stages. The first decomposition stage of GG started at 68.6 °C and ended at 168.9 °C with a loss of 10.13%, while in GG-PAAm/Er_2_O_3_ NC it started at 86.3 °C and terminated at 199.8 °C with a loss of 2.1%. The logical explanation for these results could be the moisture content loss associated with the gum. The second phase corresponding to the decomposition of sugars in GG started at 255.9 °C and terminated at 340.7 °C with a weight loss of 42%, while in GG-PAAm/Er_2_O_3_ this phase began at 264.7 °C and lasted till 342.9 °C with a weight loss of 38%. The results portrayed that the nanocomposite with Er_2_O_3_ as filler has acquired higher thermal stability with lesser mass loss than the parent gum.

**Figure 3:**
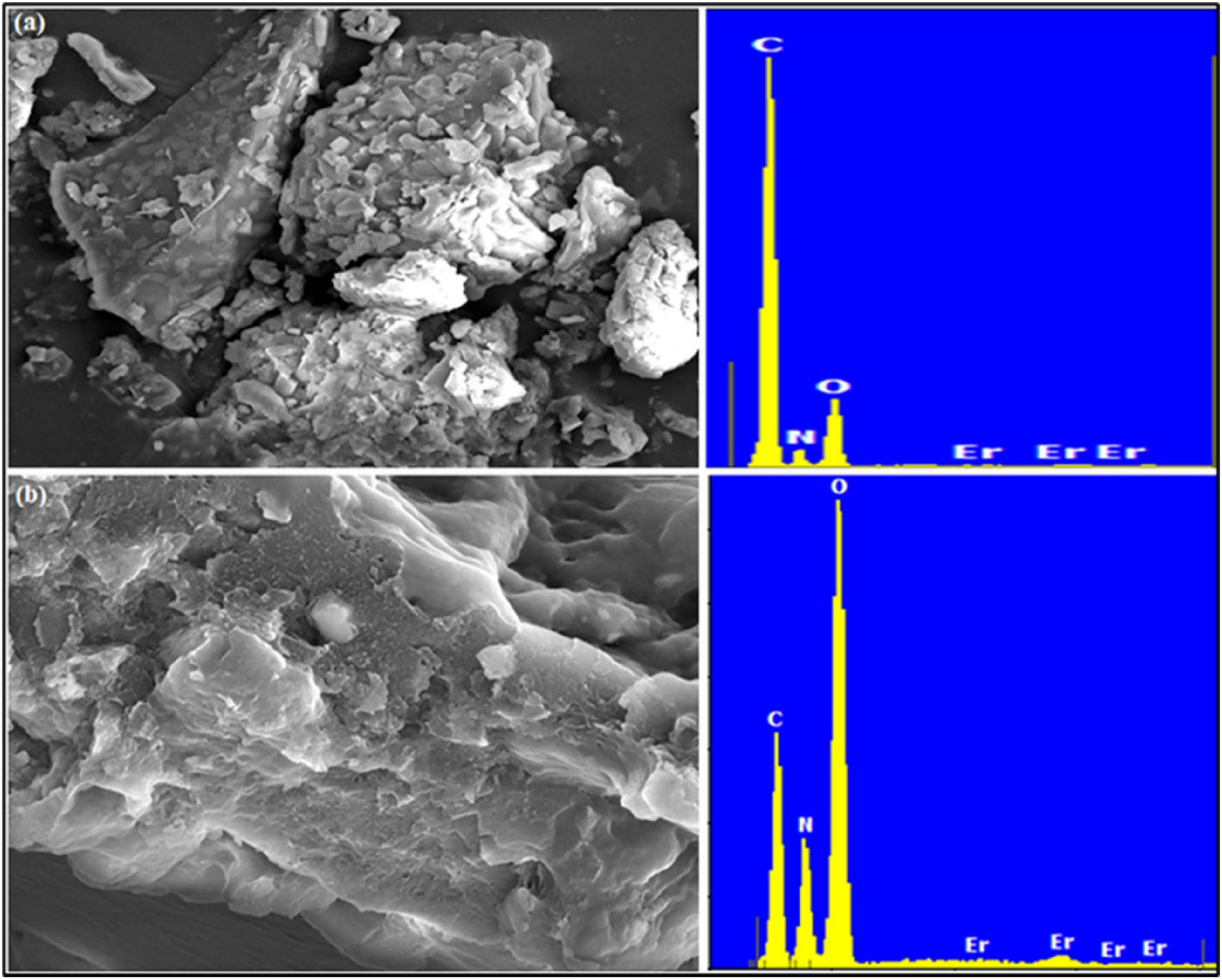
SEM micrographs of (**a**) GG-PAAm/Er_2_O_3_ nanocomposite, and (**b**) NB-loaded nanocomposite along with corresponding EDX spectra.

The SEM micrographs along with related EDX spectra of GG-PAAm/Er_2_O_3_ NC and NB-loaded GG-PAAm/Er_2_O_3_ NC are presented in Fig. 3a,b. The morphology of GG-PAAm/Er_2_O_3_ NC displayed an irregular, uneven rough surface with heterogenous porous structures probably because of the cross-linking network suitable for adsorption. The existence of sufficient pores of different sizes and shapes were primarily accountable for greater surface area and admirable adsorption efficacy of GG-PAAm/Er_2_O_3_ NC. Figure 3b of GG-PAAm/Er_2_O_3_ NC, after confiscation of NB, exhibited almost smooth texture confirming the adsorption of NB onto GG-PAAm/Er_2_O_3_ NC. The EDX spectra for corresponding SEM micrographs are also revealed in Fig. 3a,b. The existence of C, N in the EDX spectra of NB-sorbed GG-PAAm/Er_2_O_3_ NC recommended the successful confiscation of NB onto the adsorbent surface. Considerable changes in the surface morphology of GG-PAAm/Er_2_O_3_ NC occurred after the sequestration of NB. The pores nearly disappeared, which might possibly be due to the occupation and entrapment of NB molecules in the pore structures.

**Figure 4:**
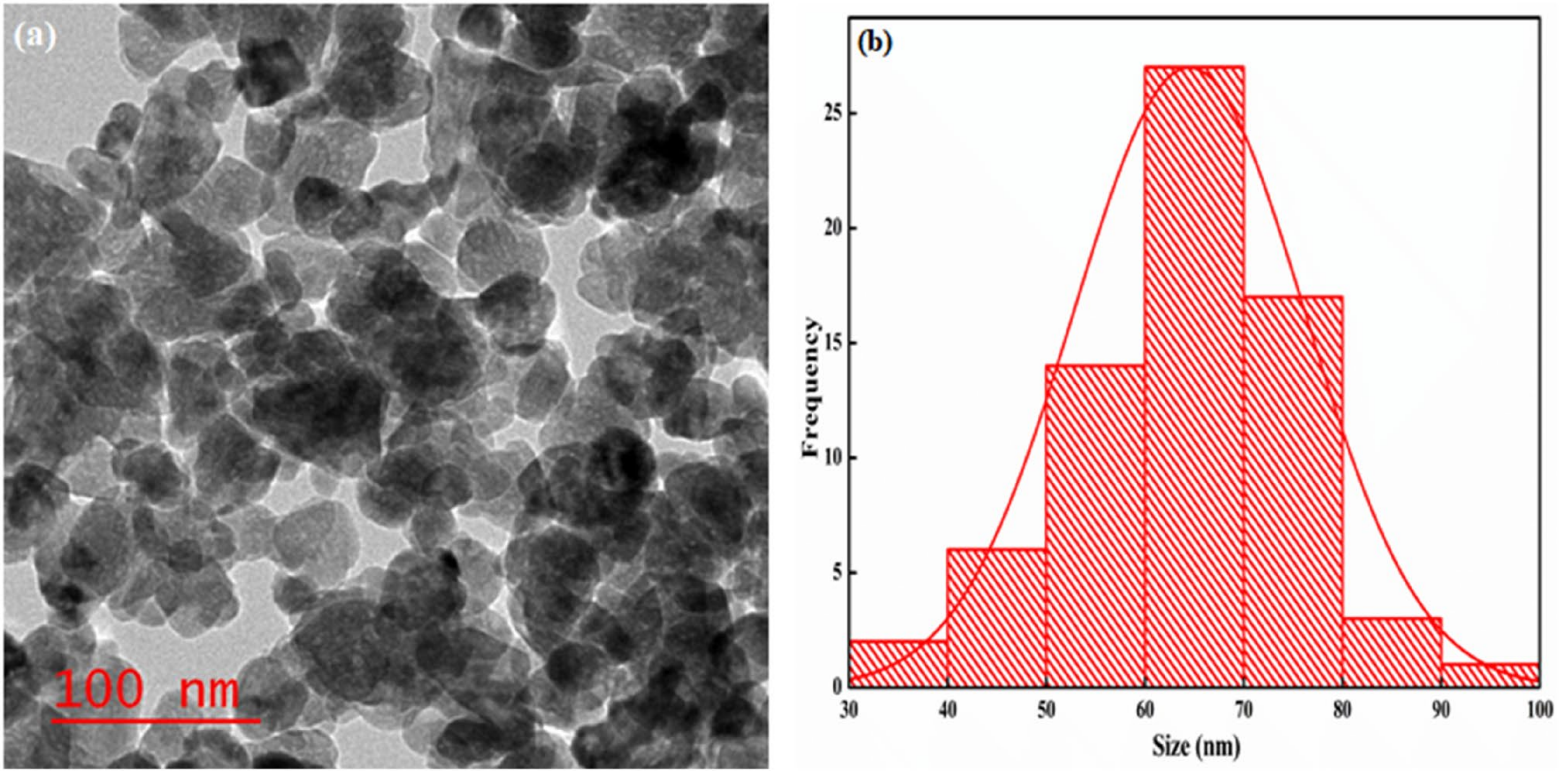
TEM micrographs of (**a**) GG-PAAm/Er_2_O_3_ nanocomposite, and (**b**) histogram.

**Figure 5:**
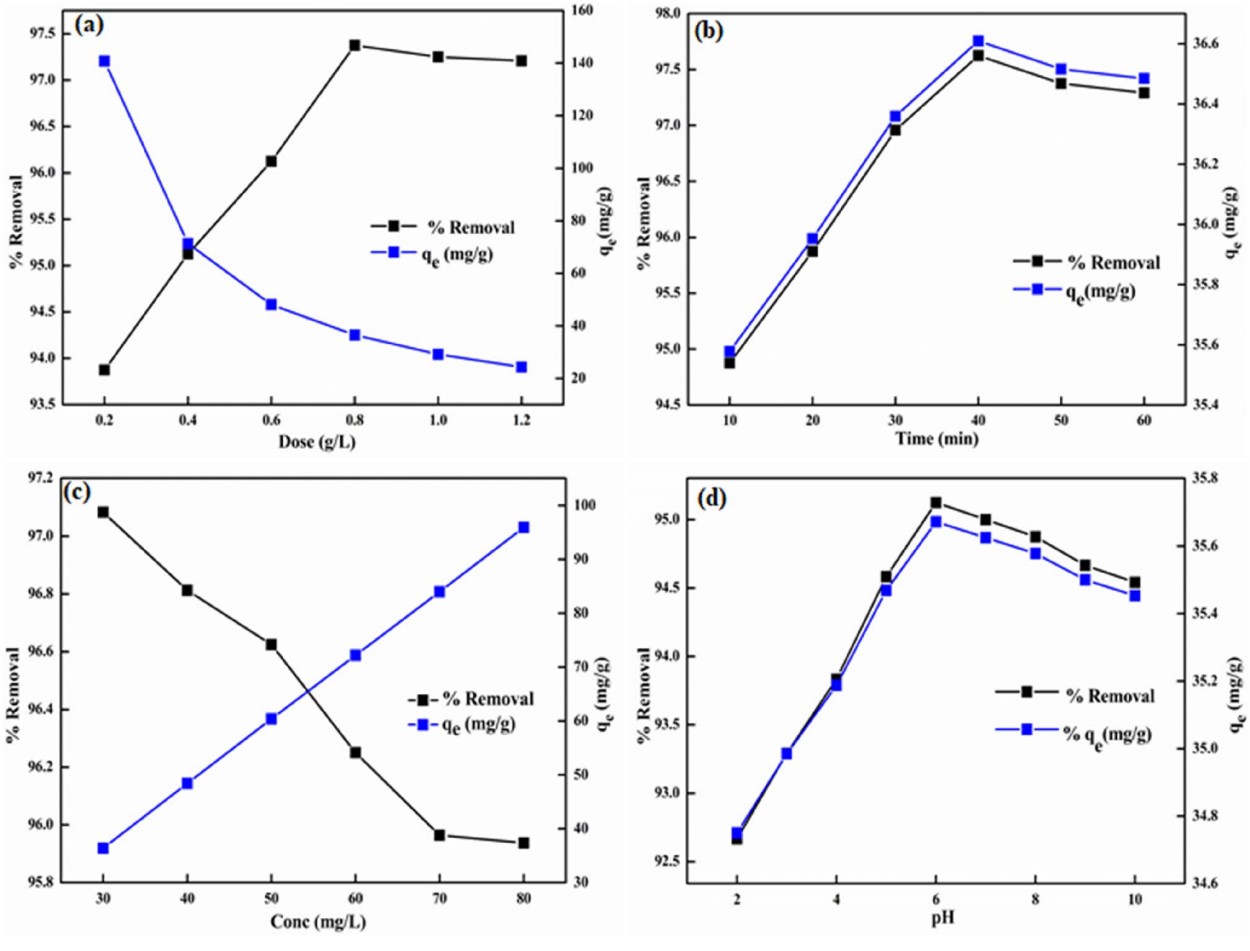
Effect of operational parameters on the removal of NB: (**a**) dose, (**b**) contact time, (**c**) initial concentration, and (**d**) initial solution pH.

To examine the structural morphology of GG-PAAm/Er_2_O_3_ NC, TEM investigation was performed. Figure 4a displays the TEM image for GG-PAAm/Er_2_O_3_ NC. The average size of GG-PAAm/Er_2_O_3_ NC was determined employing Image J software. The particle size distribution curve (Fig. 4b) suggested that the typical particles size ranged from 60 to 70 nm, that was in good agreement with XRD data. Moreover, the TEM image also confirmed the efficacious incorporation of Er_2_O_3_ within the biopolymer matrix. The grey portions in Fig. 5a demonstrated the GG and PAAm matrix, while the darker portions were accredited to Er_2_O_3_ nanoparticles randomly distributed into the GG-PAAm polymer matrix. In addition, such morphological features offered the GG-PAAm/Er_2_O_3_ NC a larger surface area.

### Effect of operational parameters on the removal of nile blue

The sorption characteristics of the nanocomposite for NB elimination was investigated in terms of different influencing parameters such as adsorbent dosage, initial NB concentration, agitation time, and initial solution pH at 303 K.

The reliance of aqueous phase removal of NB on variation in dosage was explored in 0.2–1.2 g/L range at pH 6, and the data is presented in Fig. 5a. The %R initially increased from 93.87 to 97.37 with an increment in adsorbent loading up to 0.8 g/L due to greater available active sites onto nanocomposite surface or an overall improved surface area, followed by a slow decrease thereafter. This decline in uptake efficacy on further increment in dosage might be due to higher accessible active sites relative to the number of NB molecules^54^. The removal capacity, however, showed an opposing trend with increase in adsorbent loading, which might be attributed to the overlapping of active sites, and/or decrease in surface area due to aggregation of adsorbent particles at higher dose that limited the removal capacity^54,55^. Therefore, 0.8 g/L of nanocomposite was selected as optimal dose for further studies. A similar trend of improved confiscation efficacy for NB with an increment in dosage of AC/CoFe_2_O_4_ nanocomposite^56^ and CuWO_4_ nanoparticles^57^ are described in the literature.

The agitation time is critical and significant factor in removing perilous pollutants. The effect of shaking time (10–60 min) on the sorbed NB onto the nanocomposite (0.8 g/L) is depicted in Fig. 5b. A rapid adsorption of NB occurred in the first 30 min (94.87–96.95%) suggesting a fast confiscation rate of NB molecules, gradually attaining equilibrium at 40 min with 97.62% removal. So, 40 min was preferred as the optimal equilibrium time for further studies. Initially, the removal process was rapid because of the existence of sufficient active surface-sites for absorbing NB. Subsequently, a decrement in the adsorption rate on increasing the shaking time was associated with the saturation of surface-active sites leading to decrease in the adsorption effectiveness. Similar trend in the NB removal was reported for clay/starch/MnFe_2_O_4_^58^ and CNT/MgO/CuFe_2_O_4_ magnetic composite^59^ but with relatively higher equilibrium time of 60 min and 50 min, respectively.

The impact of change in the initial NB concentration (30–80 mg/L) on removal process was examined using optimal GG-PAAm/Er_2_O_3_ NC dosage (0.8 g/L) and time (40 min) at pH 6. The obtained results, depicted in Fig. 5c, revealed that the sequestration of NB declined from 97.08 to 95.93% with increasing initial NB concentration from 30 to 80 mg/L, which could be understood in terms of two adversative effects. A fixed mass of GG-PAAm/Er_2_O_3_ NC (0.8 g/L) has a definite number of surface-active sites. At low concentrations, the surface binding sites overwhelm the feeble number of NB molecules, which resulted in higher removal efficacy. However, when the solution concentration was increased further, the NB molecules progressively occupied the vacant sites, which reduced the number of available active sites. At high concentrations, fewer dye molecules occupied the remaining surface sites, hence decrease in removal efficiency. The increment in *q*_e_ (36.40–95.93 mg/g) at higher solution concentration might be as a consequence of higher interactions between dye molecules and GG-PAAm/Er_2_O_3_ NC, or higher concentration gradient and/or increased driving force surpassing the mass transfer^60^. The optimal concentration for NB was 80 mg/L. Similar outcomes for NB adsorption with change in initial NB concentration were noticed for iron oxide nanoparticles^61^ and acrylamide- or 2-hydroxyethyl methacrylate-based copolymeric hydrogels^62^.

The solution pH is a crucial factor that plays a significant role in the removal process of contaminants. The extent of sorption is controlled by surface charge of the nanocomposite and the ionization of sorbate species that are governed by the solution pH^63^. Thus, the impact of the solution pH on NB confiscation was scrutinized in 2–10 pH range at optimum operating conditions (Fig. 5d). The pH of the solution reflects the nature of the physicochemical interactions of the NB molecules and the active sites of the GG-PAAm/Er_2_O_3_ NC. The pK_a_ value of NB is 9.27 revealing its existence in the cationic form in the studied pH range. The pH_zpc_ (= 5.6) of the nanocomposite meant that the surface was positively charged at pH < pH_*zpc*_ implying an electrostatic repulsion between positive GG-PAAm/Er_2_O_3_ NC surface and cationic NB, which might have declined the removal rate in the pH range of 2–6. However, as the %uptake was significantly affected, it implied that other type of interactions like hydrogen bonding and π–π interactions might be responsible for higher removal rate (82.43–90.6%) in the 2–6 pH range. Further increase in the pH changed the surface of nanocomposite to negative, which made electrostatic attractions to be a part of removal mechanism and the %uptake augmented to 95.66% till pH 9. After that, the dye became negative and electrostatic repulsion declined the %removal. Similar trend was depicted for NB adsorption onto MoO_3_/Ppy nanocomposite^6^.

### Adsorption equilibrium isotherms

The analyses of isothermal equilibrium data at constant temperature by applying different isotherm models provide efficacious perspective with regard to maximum sorption capacity, homogeneity or heterogeneity of the adsorbent surface, affinity of the adsorbent towards adsorbate, coverage type, energy of adsorption, and the mechanism of adsorption. The corresponding isotherm parameters were determined using *C*_*e*_ versus *q*_*e*_ plots employing Langmuir (Eq. 1), Freundlich (Eq. 2), Temkin (Eq. 3) and D-R (Eq. 4) models.

Langmuir isotherm asserts the sorption of NB onto the nanocomposite surface with finite number of energetically equivalent surface-active sites^64^ having equal affinity for NB molecules leading to monolayer formation.

1 $${q}_{e}=\frac{{Q}_{m}b{C}_{e}}{1+b{C}_{e}}$$

2 $${q}_{e}={K}_{f}{{C}_{e}}^\frac{1}{nf}$$

3 $$qe={\beta }_{t}\mathrm{ln}\left({K}_{t}{C}_{e}\right)$$

4 $${q}_{e}=({q}_{D})\mathrm{exp}[-{K}_{ad}\{\left(1+\frac{1}{Ce}\right){\}}^{2}$$

Here, *C*_*e*_ (mg/L) and *q*_*e*_ (mg/g) is the residual equilibrium NB concentration in fluid phase and the amount of NB sorbed onto the solid phase, respectively, *Q*_*m*_ (mg/g) signifies maximum adsorption efficiency of the nanocomposite required to form monolayer of sorbate on its surface, *b* (L/mol) is a Langmuir constant, *K*_*f*_ (mg/g)(L/mg)$^{{1}/{\text{n}_{\text f}}}$ is an indicator of sorption efficiency, *n*_*f*_ indicates heterogeneity of nanocomposite surface and mutual interaction between sorbed species, *1/nf* signifies functional strength of sorption, *K*_*t*_ (L/g) is binding constant associated with maximal binding energy, *β*_t_ (= RT/*b*_*t*_) is a constant related to heat of adsorption, and *q*_*D*_ (mg/g) is D–R sorption effectiveness.

The dimensionless factor, *R*_*L*_ = $\frac{1}{1+b{C}_{e}}$ is used to evaluate the feasibility and favorability of adsorption procedure.

**Figure 6:**
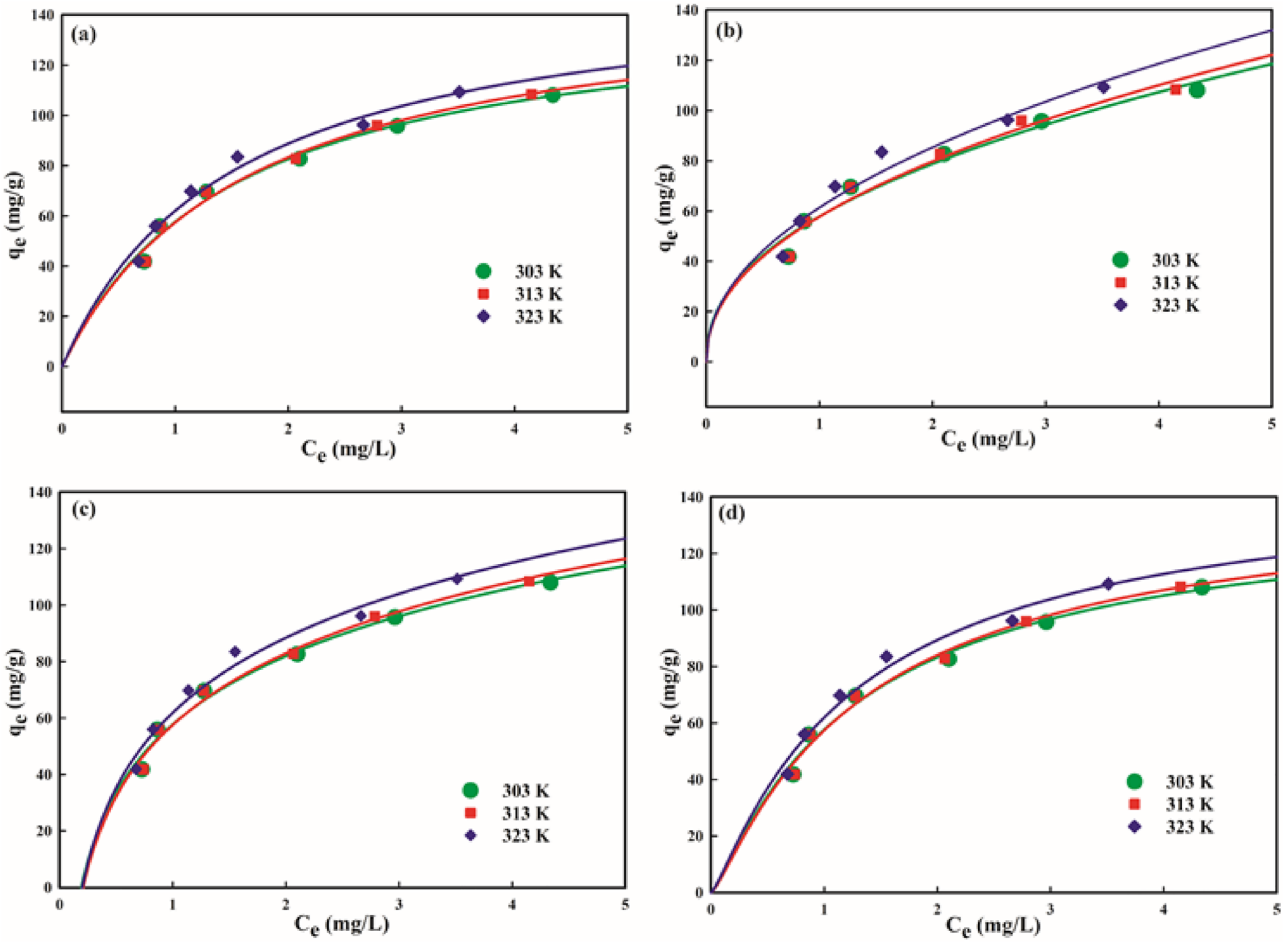
Isotherm plots: (**a**) Langmuir, (**b**) Freundlich, (**c**) Temkin, and (**d**) D-R for NB uptake.

The corresponding isotherm model parameters along with correlation coefficients (*R*^2^) and standard error of estimate (*SEE*) at the studied temperatures were estimated from *q*_*e*_ versus *C*_*e*_ curves for Langmuir (Fig. 6a), Freundlich (Fig. 6b), Temkin (Fig. 6c) and D-R (Fig. 6d) isotherms, and are tabulated in Table 1. An increment in the computed *Q*_m_ values from 195.16 to 225.88 mg/g with an increase in operating solution temperature (303–313 K) (Table 1) indicated an improvement in the adsorption aptitude of the nanocomposite at higher temperature probably resulting due to enhanced physical attachment between active binding sites and NB molecules, which designated the removal process as endothermic. The *b* parameter (0.061–0.069 L/g) varied in the order: 303 K < 308 K < 313 K, which accounted for the best NB-nanocomposite binding at higher temperature. The *R*_L_ parameter (0.375–0.193) lying between zero to unity validated the energetically favorable sorption, and contemplated a strong NB-nanocomposite interaction^14^ probably accounting for high percentage elimination of NB. The parameter, *Q*_*m*_ is used to evaluate the sorption potential of a given adsorbent. A considerably higher adsorption efficacy (*Q*_m_) of 225.88 mg NB/g at 313 K relative to most described adsorbents in the literature for NB removal (Table 2) and various tree gum-based nanocomposites for other dyes confiscation (Table 3) validated the admirable sorption effectiveness of GG-PAAm/Er_2_O_3_ NC.

**Table 1:** Isotherm parameters.

Isotherm model	Isotherm constant	Nile blue		
		303 K	308 K	313 K
Langmuir	*Q*_*m*_	195.17	209.45	225.88
	*b*	0.061	0.065	0.069
	*R*_*L*_	0.375	0.254	0.193
	*R*^*2*^	0.975	0.989	0.995
	*SEE*	0.628	0.356	0.258
Freundlich	*K*_*f*_	57.88	57.93	61.44
	*1/n*_*f*_	0.445	0.465	0.475
	*R*^*2*^	0.962	0.971	0.980
	*SEE*	1.28	2.55	4.56
Temkin	*K*_*t*_	4.84	5.21	5.46
	*b*_*t*_	0.024	0.032	0.041
	*R*^*2*^	0.975	0.981	0.985
	*SEE*	3.40	3.34	4.16
D-R	*q*_*D*_	139.74	143.85	149.77
	*E*	0.081	0.087	0.091
	*R*^*2*^	0.980	0.978	0.989
	*SEE*	3.48	3.46	4.03

**Table 2:** Comparison of *Q*_*m*_ (mg/g) values with other available adsorbents for nile blue removal.

MoO_3_/polypyrrole nanocomposite	189	6
AC/CoFe_2_O_4_ composite	86.24	56
P(AAm-co-AcA)	16.86	62
Lignocellulosic agricultural waste	94.85	63
Sulfonated phenol–formaldehyde resin	107	65
Modified activated sludge	515.1	66
Magnetically-modified natural biogenic iron oxides	50.1	67
Graphene oxide coated by polydopamine	131.58	68
Magnetically modified spent grain	44.7	69
Magnetic MWCNTs	169.49	70
GG/PAAm/Er_2_O_3_ nanocomposite	225.88	Present study

**Table 3:** Comparison of *Q*_*m*_ (mg/g) values of various tree gum-based nanocomposites and their efficiency towards various dyes.

GG/NiWO_4_ nanocomposite	Phloxine B Crystal violet	220.21 170.42	14
LBG-cl-poly(DMAAm) hydrogel	Brilliant green	142.85	71
KG-g-PMETAC/MMT	Methylene blue	155.85	72
Gum xanthan/psyllium hydrogel	Eriochrome black T	12.69	73
GG-g-PAAm)-PVA	Crystal violet	45.45	74
Gg–cl–PAA	Methylene blue	909.09	75
XG-g-PAM/SiO_2_ nanocomposite	Congo red	209.2	76
GrA-cl-poly(AAm)	Malachite green	4.76	77
GA-cl-poly(AAm)	Crystal violet	90.90	78
Almond gum	Methylene blue	500	79

Freundlich isotherm contemplates adsorption onto the surface of adsorbent with heterogeneous distribution of binding sites. It also illustrates the sorption as non-ideal and reversible phenomena where sorbent has non-uniform affinity leading to the multilayer sorption^80^. The magnitude of the parameter, *n*_*f*_ is an indicator of the sorbent surface heterogeneity, and its value close to unity expresses a higher surface heterogeneity. The value of 1/*n*_*f*_ is a measure of favorable, unfavorable or irreversible sorption process. The 1/*n*_*f*_ value < 0.5 specifies the facile sorption, 1/*n*_*f*_ > 1.0 denotes cooperative adsorption, while 1/nf > 2 depicts that NB is hardly sorbed^54^. The values 1/*n*_*f*_ below 0.5 (0.445–0.475) together with relatively higher *K*_*f*_ (57.88–61.44 (mg/g)(L/mg)^1/*n*f^) (Table 1) supported the positive and favorable sorption of NB. The increasing trend in *K*_*f*_ with rise in temperature confirmed the endothermic trait of sorption.

Temkin isotherm model is employed to investigate the interaction between sorbate and sorbent^81^. It takes into account that heat of adsorption for pollutants diminishes linearly instead of logarithmically with an increase in coverage of the nanocomposite surface. The equilibrium binding constant, *K*_t_ (L/g) values (4.84–5.46) displayed an incremental change with rise in temperature (303–313 K) pointing out a relatively enhanced electrostatic interaction between nanocomposite surface-sites and NB molecules at high temperature. An increment in the *b*_T_ values, which is related to the heat of sorption from 0.024 to 0.041 kJ/mol (Table 1) testified to slightly higher bonding probability of NB at elevated temperature (313 K). Further, the endothermic physisorption of NB was evidenced by the positive *b*_T_ values below 8 kJ/mol, which is confirmed by the pertinent thermodynamic parameter (∆*H*°)^82^.

Dubinin–Radushkevich (D–R) isotherm model adopts a pore filling sorption mechanism with multilayer character involving van der Waals interaction and is usually used to recognize the mode of adsorption, that is, physical or chemical^83^. It also provides reasonable evidence about the adsorption mechanism with possible distribution of energy onto non-homogenous surface of adsorbent. The mean free energy of adsorption (*E*) was deduced using the equation, *E* = $\frac{1}{(2\upbeta )\frac{1}{2}}$ from the value of β (mol^2^/kJ^2^), estimated from Eq. (4). If the mean free energy, *E* is 1–8 kJ/mol then physical interaction governs the adsorption mechanism, while that between 8 and 16 kJ/mol indicates ion-exchange phenomenon. However, *E* > 16 kJ/mol specifies chemical interaction. The values of *E* equal to 0.081–0.091 kJ/mol advocated physisorption. The calculated values of *q*_*D*_ (mg/g) were 139.74, 143.85, and 149.77 at 303, 308 and 313 K, which are in agreement with the similar trend in *Q*_m_ values obtained using the Langmuir isotherm plot.

The estimation of excellent fit model is described on the basis of lower *SEE* and *R*^*2*^ values close to unity. It was concluded from Table 1 that Langmuir model with *R*^*2*^ near to one (0.975–0.995) and lower *SEE* (0.628–0.258) as compared to the values recorded for other isotherm models provided the best correlation of the data. The conformity of the equilibrium data to the Langmuir model implicated that the removal procedure occurred onto homogeneous surface of nanocomposite with the formation of monolayer coverage of NB molecules.

### Adsorption kinetics

**Figure 7:**
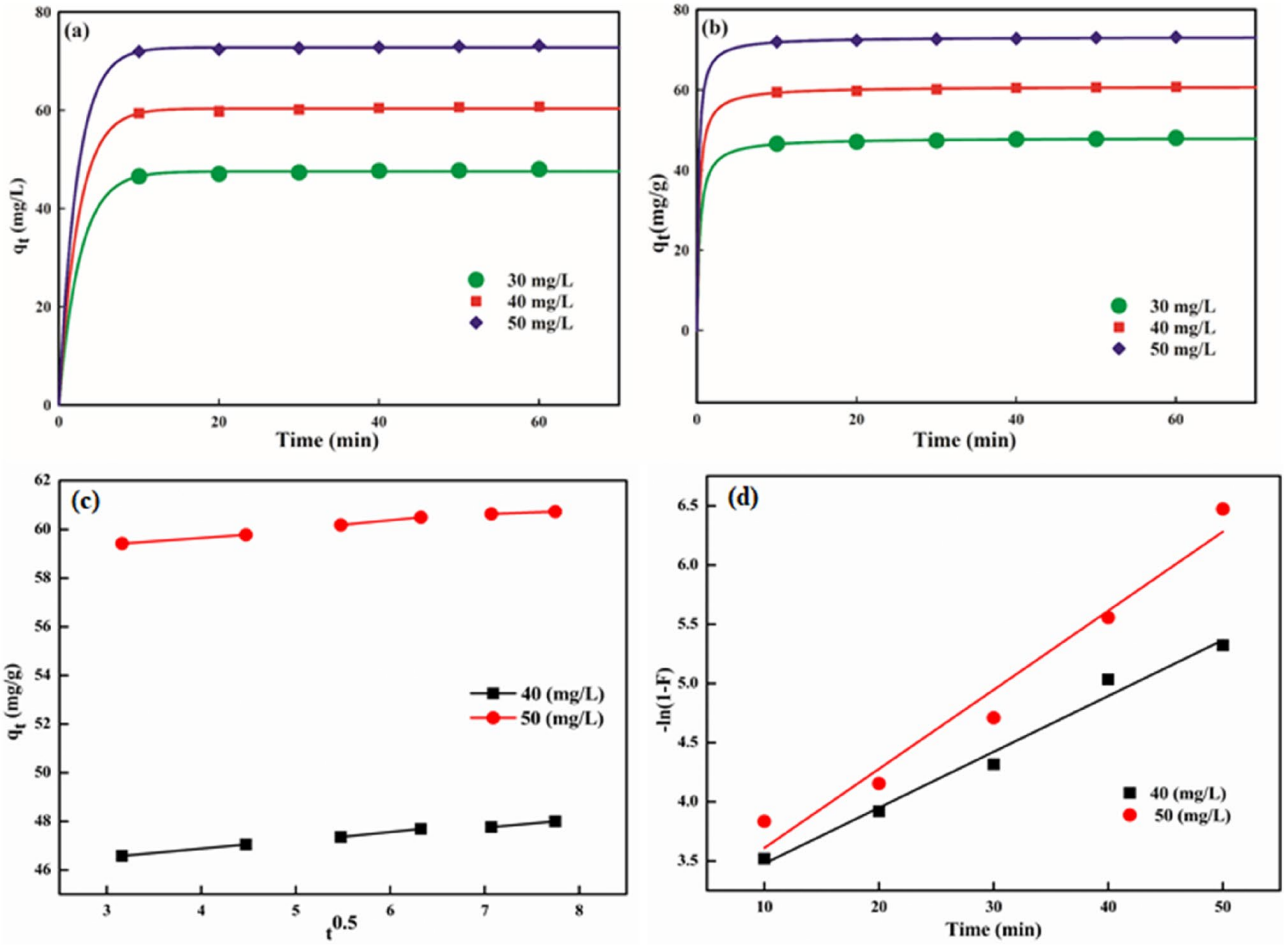
Kinetic plots: (**a**) pseudo-first order, (**b**) pseudo-second order, (**c**) intraparticle diffusion, and (**d**) liquid film diffusion for NB removal.

The adsorption kinetic models provide significant information on the rate of contaminant adsorption. These models are utilized to illustrate the experimental data to conclude the mechanism for the adsorption of contaminants from aquatic system at adsorbent–adsorbate interface. To interpret the adsorption procedure, the kinetic data was examined by pseudo-first order^84^ and pseudo-second order^85^ models by employing Eqs. (5) and (6), respectively:

5 $${q}_{t}= {q}_{e}\left(1-{e}^{-k1.t}\right)$$

6 $${q}_{t}= \frac{{\mathrm{k}}_{2}{\mathrm{q}}_{{\mathrm{e}}^{2}}.\mathrm{t}}{(1+{\mathrm{k}}_{2{\mathrm{q}}_{{\mathrm{e}}^{2}}.\mathrm{t}} )}$$

where *k*_*1*_ (1/min) and *k*_*2*_ (g/mg/min) are adsorption rate constants for pseudo-first order and pseudo-second order kinetic model, respectively. The *q*_*e*_ and *q*_*t*_ signify adsorption capability of NB at equilibrium and time *t*, respectively. The values of *k*_*1*_, *k*_*2*_ and *q*_*e*_ were determined for different initial NB concentration from the slope and intercept of the plot of *q*_*t*_ versus *t* (Fig. 7a,b), and are presented in Table 4 along with *R*^*2*^ and *SEE*. The pseudo-second order model approved the best depiction of sorption data based on higher *R*^*2*^ (0.925–0.978) and lower *SEE* values (0.077–0.136), which indicated that the removal of NB by nanocomposite was affected by the number of active binding sites instead of the initial NB concentration. The appropriateness of the pseudo-second order kinetic model in describing the experimental data recommended that the rate limiting step for NB confiscation by GG-PAAm/Er_2_O_3_ NC probably involved chemisorption mechanism. The decrease in the magnitude of *k*_*2*_ values with increment in initial NB concentration (0.069–0.059 g/mg/min) (Table 4) signified rapid adsorption at lower concentration, which could be ascribed to the lesser competition faced by NB molecules for surface active-sites suggestive of physisorption^54^.

**Table 4:** Kinetic and diffusion parameters.

[Nile blue] (mg/L)	Pseudo-first order					Pseudo-second order			
	*k*_*1*_ (1/min)	*R*^*2*^	*SEE*	*q*_*e*_(exp) (mg/g)	*q*_*e*_(cal) (mg/g)	*k*_*2*_ (g/mg/min)	*q*_*e*_(cal) (mg/g)	*R*^*2*^	*SEE*
30	0.383	0.783	0.364	47.40	47.58	0.069	48.07	0.925	0.077
40	0.412	0.891	0.382	60.04	60.38	0.062	60.86	0.965	0.093
50	0.443	0.912	0.395	72.19	72.82	0.059	73.23	0.978	0.136

	Intraparticle diffusion			Liquid film diffusion					
	*ki* (mg/gmin^0.5^)	*Ci*	*R*^*2*^	*SEE*	*k*_*D*_ (1/min)	*R*^*2*^	*SEE*		
40	0.306	45.65	0.985	0.016	0.047	0.989	0.003		
50	0.302	58.48	0.987	0.020	0.066	0.991	0.007		

Interpretation of kinetic data is crucial to conclude the adsorption procedure governing the rate controlling steps. Usually, the liquid film diffusion implicating external mass transfer of NB molecules from bulk solution to GG-PAAm/Er_2_O_3_ NC surface, intraparticle diffusion and interior pore diffusion are encompassed in the adsorptive scavenging of dyes. The Boyd liquid film and Weber-Morris intraparticle diffusion models^86,87^ are respectively expressed as Eqs. (8) and (7)

7 $${q}_{t}= {k}_{i}.{t}^{0.5}+{C}_{i}$$

8 $${\text{ln}}\left( {1 - \frac{{q_{t} }}{{q_{e} }}} \right) = k_{D} .t$$

where *k*_*i*_ (mg/g/min^0.5^) and *k*_*D*_ (1/min) are rate constants for intraparticle and liquid film diffusion, respectively and *C*_*i*_ is the intercept expressing the boundary layer thickness.

The straight-line curves of either *q*_t_ versus *t*^0.5^ (Fig. 7c) or –ln(1–*F*) versus *t* (Fig. 7d) (F = *q*_e_/*q*_t_) at 40 mg/L and 50 mg/L initial NB concentration with *C*_*i*_ = 0 delineate that the dynamics of the confiscation process of NB is controlled either by intraparticle or liquid film diffusion as the rate-limiting step. The intraparticle diffusion graphs, however, deviated from the linearity with high boundary layer contribution to the rate-controlling step (*C*_*i*_ = 45.65 and 58.48) indicating that it did not solely control the adsorption rate. Similarly, the liquid film diffusion plots also were not linear and did not pass through the origin, which precluded the liquid film diffusion as the only rate governing step. The values of *k*_*i*_ (mg/g min^0.5^) and *k*_*D*_ (1/min) were 0.306–0.302 and 0.047–0.066, respectively at the studied concentrations. It could, therefore, be inferred that the adsorption process of NB was controlled by both the diffusion mechanisms. However, based on *R*^2^ and *SEE* values for intraparticle diffusion (0.985–0.987; 0.016–0.020) and liquid film diffusion (0.989–0.991; 0.003–0.007), it could be concluded that the liquid film diffusion has a predominant role.

### Activation parameter

**Figure 8:**
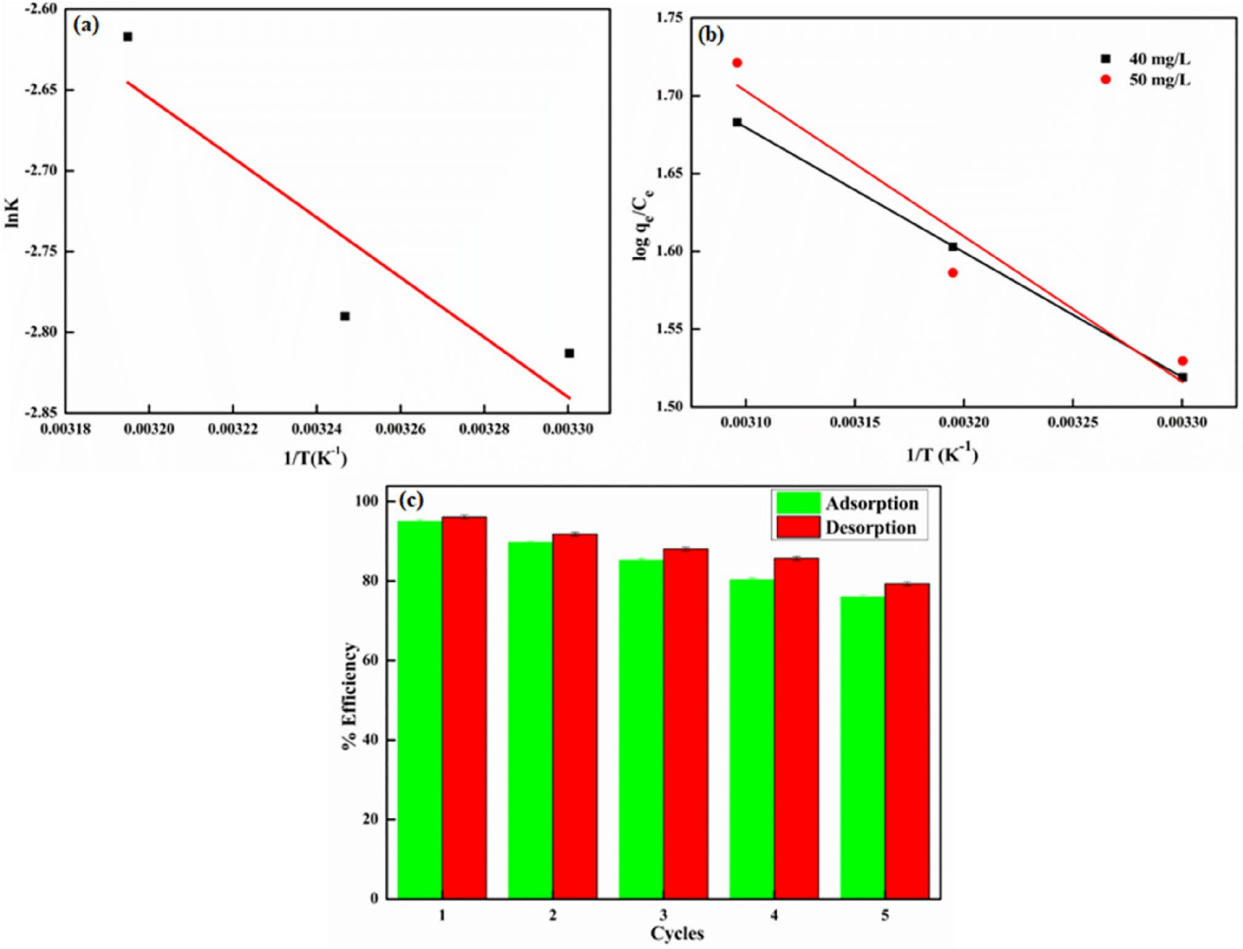
(**a**) Arrhenius plot, (**b**) van’t Hoff plot, (**c**) reusability potential of GG-PAAm/Er_2_O_3_ nanocomposite.

Arrhenius equation (ln*k*_2_ = ln*A* − *E*_*a*_/*RT*) was employed to determine the activation energy (*E*_*a*_) for the adsorption of NB onto GG-PAAm/Er_2_O_3_ NC surface, where *k*_*2*_ is the pseudo-second order rate constant. The slope of ln*k*_*2*_ versus 1/T plot, displayed in Fig. 8a, gave the precise value of *E*_*a*_*,,* and is listed in Table 5. Activation energy provides an insight about the nature of adsorption i.e., physical, or chemical. The low values of *E*_*a*_ (5–50 kJ/mol) are indicative of physisorption, whereas the *E*_*a*_ between 60 and 800 kJ/mol recommend chemisorption. Low values of *E*_*a*_ usually imply a process controlled by diffusion and greater values signify the involvement of chemical reactions. Therefore, the determined value (*Ea* = 15.33) advocated physisorption, which is consistent with the values reported in literature, which are 13.2 kJ/mol for adsorption of methyl violet onto perlite^88^ and 19.25 kJ/mol for maxilon blue 5G on sepiolite^89^.

**Table 5:** Activation energy (*E*_*a*_) for sorption of nile blue onto GG-PAAm/Er_2_O_3_ nanocomposite.

Temperature (K)	*k*_*2*_ (g/mg/min)	*R*^*2*^	*Ea *(kJ/mol)
303	0.060	0.995	15.33
308	0.061		
313	0.073		

### Thermodynamic studies

The enhancement in NB removal on increasing the temperature from 298 to 313 K represented an endothermic adsorption process. The thermodynamic parameters such as changes in free energy (Δ*G*°), entropy (*∆S*°), and enthalpy (*∆H*°) are used to determine the feasibility, spontaneity, and nature of the sorption procedure. The parameters, Δ*G*°, *∆H*°, and *∆S*° were evaluated by employing Eqs. (9) and (10):

9 $$\Delta {G}^{0} =-\mathrm{RTln}{k}_{c}= \Delta {H}^{0} -T\Delta {S}^{0}$$

10 $$\mathrm{log}{k}_{c}= - \frac{\Delta {H}^{0}}{2.303RT}+\frac{\Delta {S}^{0}}{2.303R}$$

where *k*_*c*_ = *q*_*e*_/*C*_*e*_, *R* = universal gas constant (8.314 J/mol K), and *T* = absolute temperature (K).

The slope and intercept of the linear curves of log*k*_*c*_ versus 1/*T* (Fig. 8b) provided the Δ*H*° and Δ*S*° values, respectively (Table 6). It has been reported that ∆*H°* values of 2–10 kJ/mol designates physisorption mechanism involving van der Waals interactions, between 2 and 40 kJ/mol denotes hydrogen bonding, while that above 60 kJ/mol deduces chemisorption^90^. The positive *∆H*° (15.35–17.87 kJ/mol) directed that the sorption process was endothermic and involved physisorption. Moreover, positive *∆S*° (0.079–0.087 kJ/mol/K) (Table 6) suggested an elevated randomness indicating an escalated degree of freedom at solid–liquid interface. Similarly, a decrease in the negative values of Δ*G*^◦^ (− 8.41 to − 9.67 kJ/mol) with increment in temperature advocated that the adsorption was spontaneous and more favorable at higher temperatures. It could, therefore, be concluded that the mechanism of NB sorption onto GG-PAAm/Er_2_O_3_ NC was mainly governed by physisorption at the studied temperatures (298–313 K).

**Table 6:** Thermodynamic parameters.

[Nile blue] (mg/L)	Δ*H*° (kJ/mol)	Δ*S*° (kJ/mol/K)	‒ Δ*G*° (kJ/mol)			
			298 K	303 K	308 K	313 K
40	15.35	0.079	8.41	8.81	9.21	9.61
50	17.87	0.087	8.35	8.79	9.23	9.67

### Desorption and regeneration studies

The intention of regeneration is not only to recover the removal efficacy of spent adsorbent, but also to recycle and reuse the valuable adsorbent for several series of sorption–desorption without loss of efficiency and stability, which might be helpful in sustainable management of the waste adsorbents, and would cut the overall treatment cost. Since operating solution pH had significant impact on NB confiscation by GG-PAAm/Er_2_O_3_ NC, it was essential to control pH during desorption. For regeneration investigation, 2.0 g/L of GG-PAAm/Er_2_O_3_ NC was agitated with NB solution (50 mg/L) for 1 h, then 0.1 mol/L NaOH was utilized as an eluent. Figure 8c demonstrates the extent of NB removal by GG-PAAm/Er_2_O_3_ NC up to fifth sorption–desorption cycles. After fifth cycle, the R% diminished from 96 to 79, which might be explicated to the large number of reversible surface-sites of GG-PAAm/Er_2_O_3_ NC. The regeneration results pointed out that the GG-PAAm/Er_2_O_3_ NC was recyclable and extremely effective in eliminating NB signifying the excellent potential relevance of the adsorbent at industrial scale.

### Biodegradation exploration

**Figure 9:**
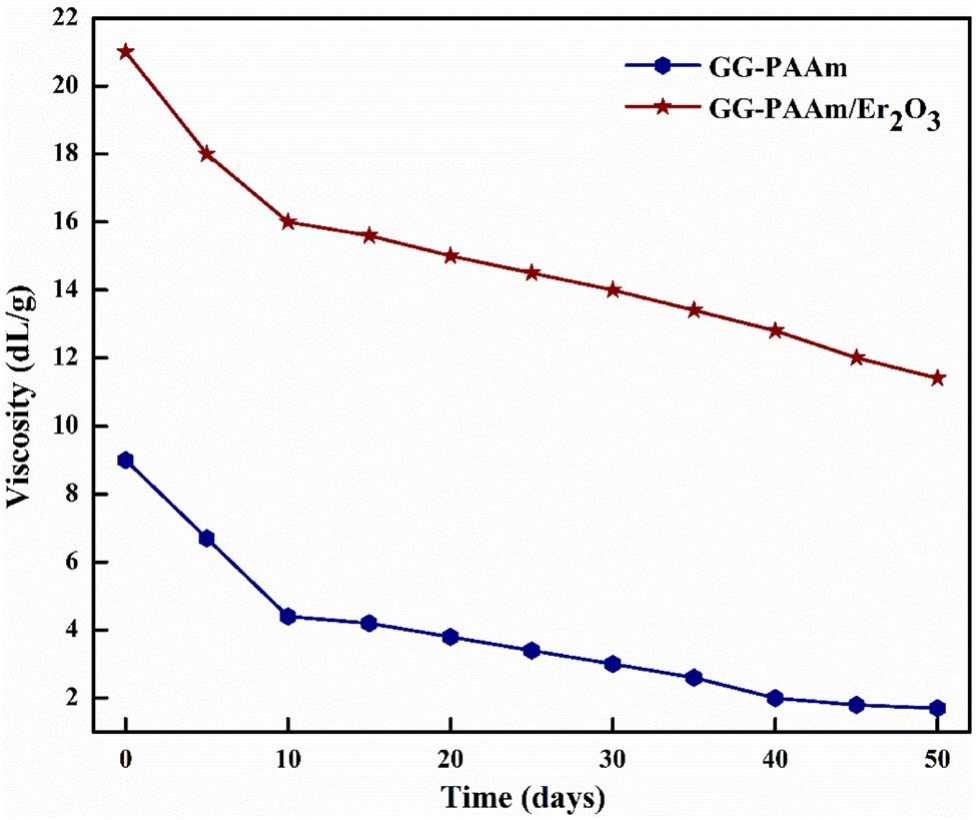
Biodegradation curves of GG-PAAm and GG-PAAm/ Er_2_O_3_ nanocomposite.

Viscometry method was used to appraise the biodegradability progress of GG-PAAm and GG-PAAm/Er_2_O_3_ NC. The advances in biodegradation was examined by assessing the intrinsic viscosity after every five days. From Fig. 9, it was apparent that both GG-PAAm and GG-PAAm/Er_2_O_3_ NC were susceptible to biodegradation. The solution depicted degradation in 5–50 days as the solution disclosed a significant loss in the viscosity, which suggested the biodegradable nature of the biopolymers. However, GG-PAAm/Er_2_O_3_ NC portrayed lesser degradability relative to GG-PAAm matrix, which might be due to an increased mechanical strength owing to erbium oxide doping.

### Mechanism of NB adsorption onto GG-PAAm/Er_2_O_3_ nanocomposite

**Scheme 1:**
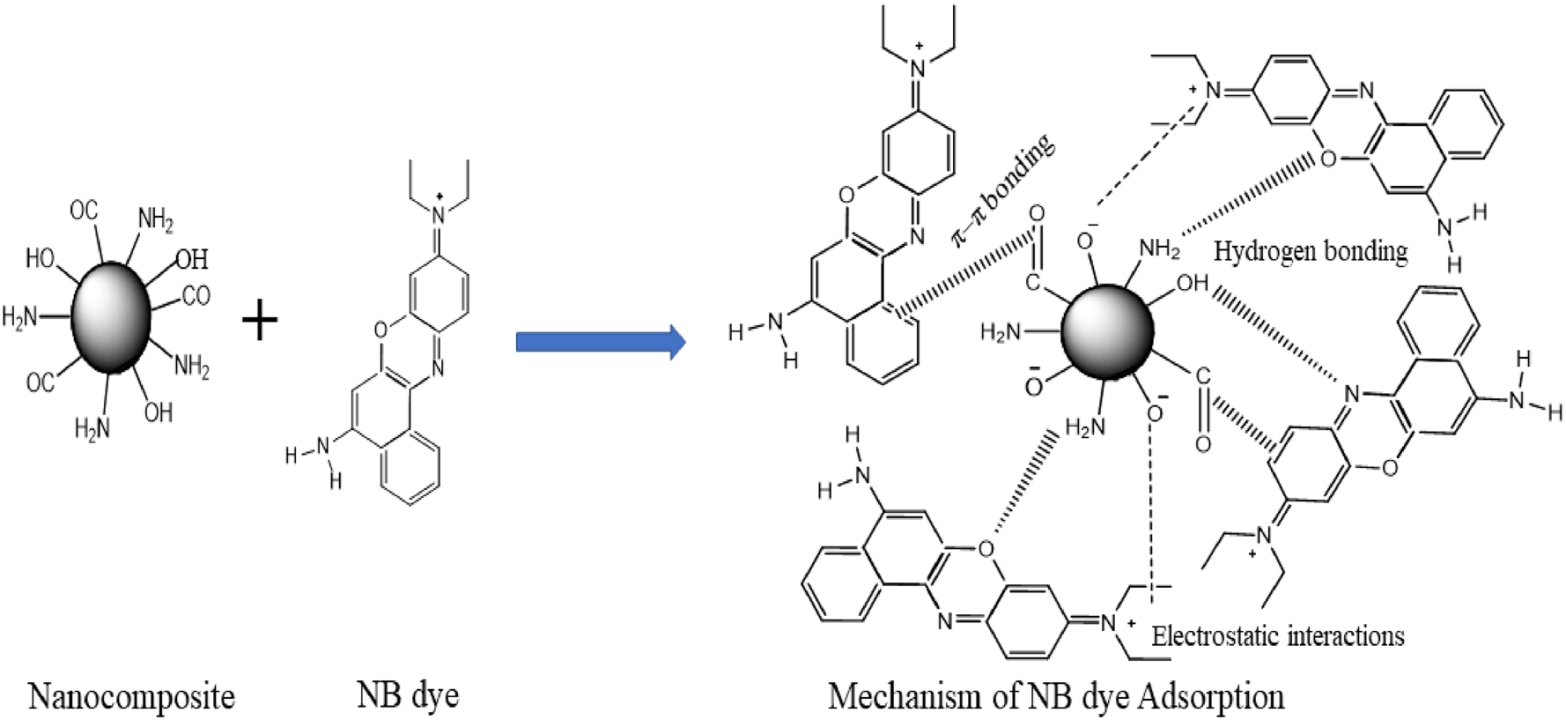
Schematic representation of the plausible interactions of NB with GG-PAAm/Er_2_O_3_ nanocomposite.

The functional groups on the surface of GG-PAAm/Er_2_O_3_ NC, and the initial pH of the dye solution play significant roles in the adsorption of NB. The adsorption of pollutants onto different adsorbents generally occurs through various interactions such as electrostatic, hydrogen bonding, dipole–dipole, van der Waals forces and π–π. To explicate the mechanism accountable for the confiscation of NB by GG-PAAm/Er_2_O_3_ NC, pH and FTIR studies were utilized. The percentage removal of NB with pH suggested electrostatic interaction as one of the mechanisms responsible for NB uptake. The slight change in the position of IR spectral peak from 3028 to 3181 cm^−1^ and change in intensity of the vibrational bands at 1654 and 1081 cm^−1^ specified that the relevant functional groups were involved in the adsorption procedure through hydrogen bonding. However, the change in the peak intensities of O–H, C–O, and C–N groups confirmed the interaction of NB with existing functional groups. It might, therefore, be inferred that electrostatic interaction, hydrogen bonding and π–π interactions were mainly involved in the adsorption of NB onto GG-PAAm/Er_2_O_3_ NC surface. The plausible mechanism of NB sequestration by the GG-PAAm/Er_2_O_3_ NC is schematically illustrated in Scheme 1.

## Conclusions

A novel GG-PAAm/Er_2_O_3_ nanocomposite with admirable adsorption capacity (225.88 mg NB/g) was efficaciously fabricated by an efficient, inexpensive, environmentally benign, and easy-to-use ultrasonic-assisted polymerization process. Fourier transform infrared spectroscopy, X-ray diffraction, scanning electron microscopy, energy dispersive X-ray, transmission electron spectroscopy, thermogravimetric analysis, specific surface area (S_BET_) and pH_zpc_ measurements were used to characterize the synthesized nanocomposite, and was successfully employed for adsorptive elimination of nile blue from liquid phase. The surface area, pore volume and pore diameter of GG-PAAm/Er_2_O_3_ NC were 70 m^2^/g, 0.024 cm^3^/g and 5.796 nm, respectively. The adsorption parameters such as dose (0.8 g/L), concentration (80 mg/L), time (40 min) and pH (6) were optimized. The equilibrium data best fitted to the Langmuir isotherm model signifying homogenous sorption of NB onto the surface of GG-PAAm/Er_2_O_3_ NC. The high *Q*_*m*_ value (225.88 mg NB/g) at 313 K validated better sorption competence of the GG-PAAm/Er_2_O_3_ NC for NB confiscation. The rate of NB sorption onto the sorbent surface was governed by pseudo-second order kinetic model with intraparticle and liquid film diffusion controlling the overall rate. The positive Δ*H°* (15.35–17.86 kJ/mol) suggested endothermic physisorption, whereas Δ*S°* (0.079–0.087 kJ/mol/K) indicated an increased randomness at the sorbent-NB solution interface. The negative Δ*G°* (− 8.41 to − 9.67 kJ/mol) governed the spontaneity and feasibility of the process. The regenerated adsorbent demonstrated good performance up to fifth cycles without much loss in efficiency, which implied that GG-PAAm/Er_2_O_3_ NC could be employed as an efficacious and potent adsorbent for cationic dyes including NB sequestration from waste water.

## Supplementary Information

Supplementary Information.

## Data Availability

The datasets used and/or analysed during the current study available from corresponding author on reasonable request.
