# Supplementary material for: Efficient liquid phase confiscation of nile blue using a novel hybrid nanocomposite synthesized from guar gum-polyacrylamide and erbium oxide
Source: Sci Rep. 2022 Aug 29;12:14656. doi: 10.1038/s41598-022-18591-0 (PMC9424225; doi:10.1038/s41598-022-18591-0)
Supplement: Supplementary file 1 — Supplementary Information. [file 41598_2022_18591_MOESM1_ESM.docx]

**Supporting Information**

**Efficient liquid phase confiscation of nile blue using a novel hybrid nanocomposite synthesized from guar gum-polyacrylamide and erbium oxide**

Daud Hussain^a^, Suhail Ayoub Khan^a^, Tabrez Alam Khan*^a^, Salman S. Alharthi^b^

^a^ Department of Chemistry, Jamia Millia Islamia, Jamia Nagar, New Delhi 110025, India

^b^ Department of Chemistry, College of Science, Taif University, P.O. Box 110999, Taif

21944, Saudi Arabia





Fig. S1: TGA plots of GG and GG-PAAm/Er_2_O_3_ nanocomposite
